# Health care utilization in patients with gout: a prospective multicenter cohort study

**DOI:** 10.1186/s12891-017-1573-6

**Published:** 2017-05-31

**Authors:** Jasvinder A. Singh, Aseem Bharat, Dinesh Khanna, Cleopatra Aquino-Beaton, Jay E. Persselin, Erin Duffy, David Elashoff, Puja P. Khanna

**Affiliations:** 1 Department of Medicine at School of Medicine, and Division of Epidemiology at School of Public Health, University of Alabama at Birmingham (UAB), Faculty Office Tower 805B, 510 20th Street S, Birmingham, 35294 AL USA; 2 Medicine Service, Birmingham VA Medical Center, Birmingham, AL USA; 3 Department of Orthopedic Surgery, Mayo Clinic College of Medicine, Rochester, MN USA; 4 University of Michigan, Ann Arbor, MI USA; 5 VA Greater Los Angeles Healthcare System, Los Angeles, CA USA; 6 University of California, Los Angeles, CA USA; 7 VA Ann Arbor Healthcare System, Ann Arbor, MI USA

**Keywords:** Gout, Utilization, Predictors, Health care utilization, Flares

## Abstract

**Background:**

All published studies of health care utilization in gout have been cross-sectional to date, and most used a patient-reported diagnosis of gout. Our objective was to assess health care utilization and its predictors in patients with physician-confirmed gout in a prospective cohort study.

**Methods:**

In a multi-center prospective cohort study of U.S. veterans with rheumatologist-confirmed gout (*N* = 186; two centers), we assessed patient self-reported overall and gout-specific health care utilization with the Gout Assessment Questionnaire (GAQ) every 3-months for a 9-month period. Comparisons were made using the student’s *t* test or the chi-square, Wilcoxon rank sum test or Fisher exact test, as appropriate. Mixed effects Poisson regression was used to assess potential correlates of gout-related health care utilization.

**Results:**

Mean age was 64.6 years, 98% were men, 13% Hispanic or Latino, 32% were African-American, 6% did not graduate high school, mean serum urate was 8.3 and mean Deyo-Charlson score was 3.1. During the past year, mean gout-related visits were as follows: rheumatologist, 1.5; primary care physician, 2 visits; ≥1 inpatient visits, 7%; ≥1 ER visits, 26%; and urgent care/walk-in visit, 33%. In longitudinal analyses, African-American race and gout flares in the last 3 months were associated with significantly higher rate ratio of gout-related outpatient visits. African-American race and lack of college education were associated with significantly higher rate ratio for gout-related urgent visits and overnight stays.

**Conclusions:**

African-American race and recent gout flares were associated with higher outpatient utilization and African-American race and no college education with higher urgent or inpatient utilization. Future studies should examine whether modifiable predictors of utilization can be targeted to reduce healthcare utilization in patients with gout.

**Electronic supplementary material:**

The online version of this article (doi:10.1186/s12891-017-1573-6) contains supplementary material, which is available to authorized users.

## Background

Gout is the most common inflammatory arthritis in adults that affects 3.9% of adult Americans, and is associated with an annual health care cost of $20 billion in the U.S. [1, 2]. Due to its association with medical co-morbidities [3, 4], frequent acute flares, and chronic symptoms including chronic pain, it is not surprising that gout is associated with poor health-related quality of life (HRQoL) [5, 6], and more health care utilization [7, 8] and costs [9, 10]. Suboptimal care for gout is common in the U.S. [11–14], which might lead to frequent gout flares, leading to subsequent increase in the utilization of urgent/emergent or inpatient care. These expenses frequently exceed those associated with outpatient health care services. Thus, gout leads to significant public health and health care cost burden.

To our knowledge, very few studies have examined the predictors of health care utilization in patients with gout. In a cross-sectional study, we found that composite gout severity, heart disease, and non-emergent health care utilization were the most significant predictors of utilization of emergency/urgent care services for gout [15]. In a national database cross-sectional study, age, male gender, lower income, private insurance and Medicaid status were associated with increased emergency department utilization in gout [7]. Very high serum urate levels and presence of tophi were associated with increased utilization and costs in elderly gout patients in a retrospective database study [10]. Previous studies were limited in several ways: study design was cross-sectional or retrospective; recall bias due to a long recall period (1 year); and the diagnosis of gout was not confirmed by a physician examination. The accuracy of diagnostic code for gout varies from 36 to 61% [16, 17] to 86% [18].

Therefore, several knowledge gaps remain. To our knowledge, no prospective studies exist for health care utilization. Additionally, the relative impact of medical comorbidity vs. gout severity on health care utilization in patients with gout is not known. Therefore, we examined health care utilization in outpatient and urgent/emergent settings and its correlates in a multicenter prospective cohort study of patients with gout at two U.S. sites.

## Methods

### Study design, patient eligibility and study enrollment

In this 9-month prospective cohort study, patients 18 years of age or older at the Veteran affairs (VA) Greater Los Angeles Healthcare System and Birmingham VA Medical Center, with diagnosis of gout were identified from the medical records at these respective medical centers. Potentially eligible patients were contacted via phone and asked about their willingness to participate in the study. Those willing to participate were invited to the medical center and provided written informed consent. Four visits per participant were scheduled at every 3-month interval. Many patients were also recruited from VA community-based outpatient clinics, outpatient primary care, rheumatology and other specialty clinics at the two sites. Inclusion criteria were age between 18 and 85 years, physician diagnosis of gout, meeting the 1977 preliminary classification criteria for gout [19] and the ability to provide informed consent. All patients included in the study signed the consent forms. The Institutional review boards (IRBs) of the Birmingham VA and Greater Los Angeles Healthcare System approved the study.

### Study assessments and data analysis

Study assessments included a paper-based patient survey, physician examination and physician questionnaire. We recruited 112 patients at the VA Greater Los Angeles Healthcare System and 74 patients at the Birmingham VA. We assessed patient self-reported health care utilization with two surveys administered every 3 months: overall utilization for the last 3-months (3 months prior to each survey) with the University of California at San Diego (UCSD) Health Care Utilization (HCU) questionnaire (short recall; Additional file 1); and gout-specific health care utilization in the last year (1 year prior to each survey) with the Gout Assessment Questionnaire (GAQ) [20] Both questionnaires are copyrighted and are available from the developer on request; in addition a copy of the GAQ questionnaire was previously published as an appendix in the original publication for this questionnaire [21]. The USCD HCU asks questions about the number of outpatient visits, emergency room/urgent care visits, inpatient visits, outpatient surgery, use of medication supplies/equipment, use of ambulance, prescription and non-prescription medicines and major medical expense. The GAQ questionnaire includes questions about overall concern for gout, gout medication side effects, unmet gout need, well being during the attack and gout concern during the attack [21]. Since more than a third of veterans utilize health care from non-VA settings [22–25], we decided a priori to use patient-reported gout health care utilization from all sources, rather than using VA claims data for only VA healthcare utilization. A rheumatologist assessed each patient for gout diagnosis, and assessed the presence or absence of tophi, and their location.

Demographic and clinical characteristics were compared using the *t*-test or Wilcoxon Rank Sum test for continuous variables depending on the normality of the data distribution. Categorical variables were compared with chi-square test where *n* > 5 and Fisher exact test where *n* ≤ 5. Overall and gout-related health care utilization were described as the number and percent of patients with at least one visit, and as the mean (standard deviation [SD]) observed visits.

Mixed effects Poisson regression was used to assess potential predictors of the two gout-related health care utilization measures: (1) Number of Outpatient Visits: Rheumatology, primary care, nurse practitioner (NP) or physician assistant (PA); and (2) Number of Urgent/overnight Visits: Walk-in or urgent care clinic, emergency room at a hospital, or hospital overnight stay. Time was not included as a covariate in the model since we do not expect any particular time trend in the study as there were no population-level interventions being utilized.

Predictors in the models were selected based on their clinical relevance. Patients’ age, race (African-American vs. White vs Other), education (No college vs. some college or higher), baseline serum urate level, Charlson comorbidity index (using claims data), and gout attack in the past 3 months were included as predictors. These longitudinal models used outcomes and predictors collected at each of the four study time points: Baseline, 3, 6 and 9 months. Many of the predictors are subject level characteristics (age, race, education, baseline serum urate level and baseline Charlson index). We also included one time varying predictor: the number of gout attacks in the prior 3-month period. The outcome in these models also refers to the prior 3-month period (utilization). Therefore, the only time varying factors are measured during the same concurrent period at each of the four time points of evaluation. A random intercept for each subject was included in the model to account for the correlation within subject. Since this study used multiple time points per subject, we describe it as a repeated measures model. It is not a prognostic model per se since we are using a predictor that is measured concurrently with the outcome.

Additional cross-sectional analyses were performed (using the same two outcome measures) using only the baseline patient survey using the baseline outcomes and predictors. These models used the standard Poisson regression models using the same set of predictors described above. We also performed exploratory analyses analyzing the main outcomes by baseline gout flares in the last year, dichotomized at 2 or fewer flares in the last year versus more than 2 flares, using the longitudinal analyses. Missing data were considered missing at random, and not imputations were done.

All analyses were conducted using SAS software v9.4 (SAS Institute Inc., Cary, NC, USA) and *p*-values <0.05 were considered statistically significant.

## Results

### Patient characteristics

Of the 205 patients screened for the study, 186 patients were enrolled in the study across the two study sites (failed screening, *n* = 1; withdrew consent, *n* = 5; withdrawn by PI [did not meet ACR gout classification criteria], *n* = 13). Patients not enrolled in the study (*n* = 19) were all men with a mean age of 61.6 years; no other characteristics were available on these patients. Baseline characteristics of enrolled patients are described in Table 1. The majority of the patients were male (97.85%), with a mean age of 64.6 ± 10.9 years. Sixty-four percent of the patients had at least some college level education. Twenty-one percent had tophi, mean serum urate was 8.3 and mean physician and patient gout severity were 3.1 and 5.7, respectively (Table 1). Comorbidities were common, including hypertension (81.4%) and diabetes (36.2%; Additional file 2). The extent of missing data ranged 13–14% for the two outcomes of interest.

**Table 1:** Baseline characteristics

	Total Cohort *N* = 186	Los Angeles *N* = 112	Birmingham *N* = 74
Age, Mean (SD)	64.6 (10.9)	65.7 (11.2)	62.9 (10.2)
Gender, *N* (%)			
*Male*	182 (97.85)	109 (97.32)	73 (98.65)
*Female*	4 (2.15)	3 (2.68)	1 (1.35)
Race, *N* (%)			
*American Indian*	1 (0.54)	1 (0.89)	0 (0)
*Asian*	6 (3.23)	6 (5.36)	0 (0)
*Native Hawaiian or other Pacific Islander*	1 (0.54)	1 (0.89)	0 (0)
*Black or African American*	60 (32.26)	17 (15.18)	43 (58.11)
*White*	107 (57.53)	76 (67.86)	31 (41.89)
*Other*	11 (5.91)	11 (9.82)	0 (0)
Ethnicity, *N* (%)			
*Hispanic or Latino*	21 (13.04)	20 (18.69)	1 (1.85)
*Not Hispanic or Latino*	140 (86.96)	87 (81.31)	53 (98.15)
*Missing*	25	5	20
Education, *N* (%)			
*Grades 9 to 11*	11 (5.98)	8 (7.27)	3 (4.05)
*High school graduate*	35 (19.02)	17 (15.45)	18 (24.32)
*Some college*	88 (47.83)	54 (49.09)	34 (45.95)
*College graduate*	30 (16.30)	13 (11.82)	17 (22.97)
*Beyond college*	20 (10.87)	18 (16.36)	2 (2.70)
Tophi Diagnosed, *N* (%)			
*No*	136 (78.61)	74 (67.89)	62 (96.88)
*Yes*	37 (21.39)	35 (32.11)	2 (3.13)
*Missing*	13	3	10
Serum Uric Acid, Mean (SD) [Median (IQR)]	8.3 (3.4) [7.9 (3.5)]	7.5 (1.9) [7.5 (3.0)]	10.1 (5.1) [9.7 (8.2)]
Severity of Gout, Mean (SD)			
*Patient Assessment (GAQ)*	5.7 (3.1) [6.0 (5.0)]	6.1 (3.1) [6.5 (4.5)]	5.2 (3.1) [4.0 (5.0)]
*Physician Assessment*	3.1 (2.7) [3.0 (4.0)]	3.0 (2.5) [3.0 (3.5)]	3.3 (2.9) [2.8 (5.0)]
Charlson Comorbidities, Mean (SD)	5.14 (2.74)	5.36 (3.02)	4.75 (2.16)

α = *T*-test; β = Fisher Exact test; γ = Wilcoxon test; ε = Chi-square test

*Abbreviations*: *IQR* inter-quartile range, *SD* standard deviation, *GAQ* Gout assessment questionnaire

**Table 2:** Longitudinal Multivariable-adjusted predictors of gout-related outpatient and Urgent/emergent/overnight visits^a^ in the 3 months prior to baseline

	Incidence rate ratio (95% CI)	*P*-value
Gout-related Outpatient visits		
Age (for every 1 year increase in age)	1.00 (0.99, 1.01)	0.49
African American (ref, Caucasian)	**1.80 (1.30, 2.50)**	**<0.01**
College Education (ref, no college education)	0.97 (0.81, 1.17)	0.77
Baseline sUA (for every 1 mg/dl increase)	0.98 (0.94, 1.02)	0.30
Charlson Index (for every unit increase)	0.95 (0.90, 1.01)	0.10
Gout attack in Past 3 months (ref, no Attack)	**1.28 (1.11, 1.49)**	**<0.01**
Gout-related Urgent/emergent/overnight visits^a^		
Age (for every 1 year increase)	1.01 (0.99, 1.02)	0.53
African American (ref, Caucasian)	**2.68 (1.48, 4.86)**	**<0.01**
College Education (ref, no college education)	**0.67 (0.48, 0.92)**	**0.01**
Baseline sUA (for every 1 mg/dl increase)	1.03 (0.96, 1.12)	0.41
Charlson Index (for every unit increase)	0.96 (0.87, 1.07)	0.48
Gout attack in past 3-months (ref, no Attack)	1.19 (0.84, 1.69)	0.33

^a^includes walk-in-urgent-care-visits, emergency-room-visits, hospital-over-night-visits; Bold represents statistically significant *p*-values <0.05

### Unadjusted healthcare utilization: overall and gout-related

Overall health care utilization: There were a mean of 3.9 visits to the health care provider and 1.7 telephone calls to the provider or medical staff in the past 3 months (Additional file 3). Ninety-two percent of patients had outpatient visit, 17% had inpatient visit, 41% had ER/urgent care/triage center visit and 16% had an outpatient procedure or surgery, in the past 3-months.

Gout-specific health care utilization: Seventy-one percent patients had seen primary care physician for their gout, while 53% saw a rheumatologist. During the past year, patients had a mean of 2 and 1.5 visits to primary care physician and rheumatologist, respectively (Additional file 3). Ten percent were hospitalized in the past year related to gout and 26% had ER visits related to gout (Additional file 3).

### Cross-sectional and longitudinal predictors of gout-related healthcare utilization

In cross-sectional analyses, older age, African-American race, college education and gout attack in the past 3 months were each associated with significantly higher rate ratio of gout-related outpatient visits; higher Charlson index scores at baseline were associated with lower rate ratio of gout-related outpatient visits among patients with gout (Additional file 4). African-American race and gout attack in the past 3 months were each associated with significantly higher rate ratio of gout-related urgent/overnight visits at similar period among patients with gout (Additional file 4).

In longitudinal analyses, we found that African-American race and gout flares in the last 3 months were associated with significantly higher rate ratio of gout-related outpatient visits in patients with gout (Table 2). African-American race and no college education were associated with significantly higher rate ratio for gout-related urgent visits and overnight stays (Table 2).

### Exploratory Subgroup Analyses by the number of gout flares in the last year

Patients with 2 or fewer gout flares in the last year: African-American race and gout flares in the last 3 months were associated with significantly higher rate ratio of gout-related outpatient visits and gout flares in the last 3 months with gout-related urgent visits and overnight stays (Table 3).

**Table 3:** Subgroup analyses by number of prior gout flares in the baseline period: Longitudinal Multivariable-adjusted predictors of gout-related outpatient and Urgent/emergent/overnight visits^a^ by baseline gout flares (2 or fewer flares vs. >2 flares in the last year)

A. Patients with 2 or fewer gout flares in the last year (*n* = 71 patients)		
Gout-related Outpatient visits		
Age (for every 1 year increase in age)	1.00 (0.99, 1.02)	0.53
African American (ref, Caucasian)	**2.51 (1.12, 5.64)**	**0.03**
College Education (ref, no college education)	1.47 (0.92, 2.34)	0.10
Baseline sUA (for every 1 mg/dl increase)	0.95 (0.88, 1.03)	0.22
Baseline Charlson Index (for every unit increase)	0.94 (0.85, 1.04)	0.23
Gout attack in Past 3 months (ref, no Attack)	**1.65 (1.27, 2.15)**	**<0.01**
Gout-related Urgent/emergent/overnight visits^a^		
Age (for every 1 year increase)	1.00 (0.97, 1.03)	0.87
African American (ref, Caucasian)	3.10 (0.79, 12.15)	0.10
College Education (ref, no college education)	0.66 (0.22, 1.94)	0.45
Baseline sUA (for every 1 mg/dl increase)	1.08 (0.94, 1.23)	0.28
Baseline Charlson Index (for every unit increase)	0.93 (0.78, 1.10)	0.37
Gout attack in past 3-months (ref, no Attack)	**2.60 (1.27, 5.32)**	**<0.01**
B. Patients with >2 gout flares in the last year (*n* = 110 patients)		
Gout-related Outpatient visits		
Age (for every 1 year increase in age)	1.00 (0.99, 1.02)	0.44
African American (ref, Caucasian)	1.38 (0.94, 2.01)	0.10
College Education (ref, no college education)	0.86 (0.70, 1.05)	0.14
Baseline sUA (for every 1 mg/dl increase)	1.01 (0.96, 1.07)	0.66
Baseline Charlson Index (for every unit increase)	0.95 (0.88, 1.03)	0.18
Gout attack in Past 3 months (ref, no Attack)	1.03 (0.86, 1.25)	0.73
Gout-related Urgent/emergent/overnight visits^a^		
Age (for every 1 year increase)	1.01 (0.99, 1.04)	0.23
African American (ref, Caucasian)	2.39 (1.09, 5.24)	0.03
College Education (ref, no college education)	**0.61 (0.43, 0.86)**	**<0.01**
Baseline sUA (for every 1 mg/dl increase)	1.04 (0.93, 1.17)	0.49
Baseline Charlson Index (for every unit increase)	0.98 (0.83, 1.15)	0.81
Gout attack in past 3-months (ref, no Attack)	0.76 (0.49, 1.18)	0.22

^a^includes walk-in-urgent-care-visits, emergency-room-visits, hospital-over-night-visits; Bold represents statistically significant *p*-values <0.05

Patients with more than 2 gout flares in the last year: People with no college education were more likely than those with college education to have gout-related urgent visits and overnight stays (Table 3).

## Discussion

In this multicenter prospective cohort study of 186 gout patients, we examined the overall health care utilization in the last 3-months and gout-related health care utilization in the last year. The assessment of both overall and gout-related health care utilization in a prospective multicenter cohort study, measurement of gout severity and confirmation of a diagnosis of gout by physical examination by a rheumatologist are significant advances with this study. We believe this is the first comprehensive, multicenter, prospective cohort study of patients with validated diagnosis of gout. Our study results add to the current literature, as discussed below.

In this predominantly male gout cohort, we found that gout-related outpatient healthcare utilization was higher in patients who were African-American, in both cross-sectional and longitudinal analyses. In a recent abstract, compared to Caucasians, African-Americans had a 2.6-times higher rate of emergency room visits/hospitalizations for gout [26]. In a previous study, Asians, but not African-Americans were more likely to seek care for gout compared to Caucasians [27]. Differences in these results may be due to differences in study design (retrospective vs. prospective cohort), study methodology (diagnostic code vs. confirmation by physician examination) and confounders adjusted (age and sex vs. age, sex, education, baseline serum urate, medical comorbidity). A higher utilization of outpatient clinic by African-Americans with gout in our study indicates that this group is accessing care for gout more often than Caucasians. However, we lacked information to assess whether this is optimal utilization or under- or over-utilization amongst African-Americans in our study. Future studies that can assess gout quality indicators can investigate this gap and offer new knowledge about health care utilization among African-American patients with gout

African-American race was associated with an statistically significant increase in the ratio for urgent/emergent/inpatient care for gout in the longitudinal analyses. We noted that even in analyses that adjusted for baseline serum urate and gout flares in the last 3 months, a significantly higher rate ratio of 2.7 was noted for African-American race. This is similar to the previously reported 2.6-times higher rate of emergency room visits/hospitalizations for gout in African-Americans [26]. More studies are needed to better understand the reasons for higher emergency/inpatient health care utilization in African-Americans with gout.

Our study identified other correlates of outpatient and urgent/emergent/inpatient care for gout. Higher education level was associated with lower utilization of gout-related urgent/emergent/inpatient care visits. This is an interesting finding, since higher education level is usually associated with better health insurance and access to healthcare system [28], which usually allows people to have easier access to outpatient clinics. It is possible that these people access healthcare for gout flares more often as an outpatient, rather than in the emergency room, thereby leading to a lower risk of emergent/inpatient care for gout flares. Another possibility is whether higher education level is associated with healthier lifestyle, better medication adherence and/or better health literacy, all of which might potentially reduce the utilization of high-cost emergent/inpatient care. This question requires further study. Considering the high cost of inpatient care compared to outpatient care, it is very desirable to manage most gout in outpatient settings.

Our finding of association of gout flares in the last 3 months with higher outpatient visits mirrors similar findings in previous database study of association of gout severity (tophi) with gout utilization [10] or composite gout severity index with urgent/emergent utilization [15].

It is not surprising that 71, 53 and 31% of patients had seen primary care physician, rheumatologist or nurse practitioner/physician assistant, respectively, for their gout in the last year, since recruitment was done from the both the primary care and rheumatology clinics. This cohort study data add to the current knowledge.

Our study findings should be interpreted considering the following limitations. Since we recruited patients from primary care and/or rheumatology clinics, patients with milder disease or with minimal comorbidities who do not see their physicians frequently may have been missed, skewing our results to reflect findings among patients with more symptomatic disease. In addition, our results for utilization were based on patient self-report, which is subject to recall bias. These findings are not generalizable to women with gout, since the sample was predominantly male, representative of the US veteran population. Study design limitations include residual confounding, despite our attempt to include several important potential confounders of health care utilization, and type II error, i.e., missing some significant effect due to small sample size. Veterans have a higher medical comorbidity load than the age-matched U.S. general population [29], therefore our findings may be generalizable to gout patients with higher comorbidities only, not the general US population. We considered data missing at random for the two outcomes of interest, thus its impact on the findings is unclear; however, the extent of missingness was 13–14%, therefore, we believe it likely had minimal impact. We considered using VA claims data for utilization outcomes. However, we were interested in assessing all gout-related healthcare utilization, not just VA health care utilization, since more than a third of veterans utilize health care from non-VA settings [22–25]. Patient self-report of gout-related utilization could better capture all gout-related utilization rather than VA claims data. We did not instruct patients as to whether they should include study visits while they counted the number of gout-related visits. It is possible that some patients may have included study-related visits; however, this would only impact the outpatient visits and not other utilization outcomes. Due to limited resources, data about the use of urate-lowering drugs were not collected, which could have offered additional insights into healthcare utilization. Future studies should assess these as potential predictors.

There are several study strengths. This was a prospective multicenter cohort study with a high response rate. Our study included two geographically different regions of the U.S., i.e., Birmingham and Los Angeles. We confirmed the gout diagnosis by a rheumatologist’s examination.

## Conclusions

In conclusion, this multicenter cohort study is among the first prospective studies to examine health care utilization in patients with gout. We described patterns of health care utilization by patients with gout in the U.S., both overall as well as gout-related utilization. We also identified several correlates of gout-related outpatient and urgent/emergent/inpatient health care utilization. Measures of gout severity (flares), as well as socio-demographic factors (race, education) were associated with overall health care utilization by patients with gout. Specifically, African-American race and gout flares in the last 3 months were associated with significantly higher gout-related outpatient utilization; African-American race and no college education were associated with significantly higher rate of gout-related urgent visits and overnight stays. Future studies need to examine whether targeting gout disease severity (modifiable predictor) by more optimal management, or improving patient health literacy with education tools or patient access to outpatient gout care by making it more affordable and accessible, can reduce high-cost health care utilization for gout, especially in the urgent/emergent care settings.

## Additional files

Additional file 1: University of California at San Diego (UCSD) health care utilization questionnaire. Description: This file shows the copyrighted UCSD health care utilization questionnaire. (DOCX 992 kb)

Additional file 2: Baseline Comorbidity Characteristics. Description: This file shows the detailed baseline comorbidity characteristics for the entire cohort and by each site for the study cohort. (DOCX 29 kb)

Additional file 3: Unadjusted estimates of overall healthcare utilization and gout-specific utilization. Description: This file shows the unadjusted estimates of overall healthcare utilization and for gout-specific utilization in the study cohort, detailed by the type of healthcare utilization. (DOCX 18 kb)

Additional file 4: Cross-sectional Multivariable-adjusted predictors of gout-related outpatient and Urgent/emergent/overnight visits in the 3 months prior to the baseline. Description: This file shows the multivariable-adjusted analyses using the cross-sectional data for both gout-related outpatient and non-outpatient healthcare utilization. (DOCX 18 kb)

## Abbreviations

GAQ: Gout assessment questionnaire
HCU: Health care utilization
SD: Standard deviation
UCSD: University of California at San Diego
VA: Veterans Affairs

## References

[1] Zhu Y, Pandya BJ, Choi HK (2011). Prevalence of gout and hyperuricemia in the US general population: the National Health and Nutrition Examination Survey 2007–2008. Arthritis Rheum.

[2] Wertheimer AI, Morlock R, Becker MA (2013). A Revised Estimate of the Burden of Illness of Gout. Curr Ther Res.

[3] Macfarlane LA, Kim SC (2014). Gout: a review of nonmodifiable and modifiable risk factors. Rheum Dis Clin North Am.

[4] Mcadams-Demarco MA, Maynard JW, Baer AN, Coresh J (2012). Hypertension and the risk of incident gout in a population-based study: the atherosclerosis risk in communities cohort. J Clin Hypertens (Greenwich).

[5] Roddy E, Zhang W, Doherty M (2007). Is gout associated with reduced quality of life? a case-control study. Rheumatology (Oxford).

[6] Singh JA, Strand V (2008). Gout is associated with more comorbidities, poorer health-related quality of life and higher healthcare utilisation in US veterans. Ann Rheum Dis.

[7] Garg R, Sayles HR, Yu F, Michaud K, Singh J, Saag KG, Mikuls TR (2013). Gout-related health care utilization in US emergency departments, 2006 through 2008. Arthritis Care Res.

[8] Hanly JG, Skedgel C, Sketris I, Cooke C, Linehan T, Thompson K, van Zanten SV (2009). Gout in the elderly—a population health study. J Rheumatol.

[9] Saseen JJ, Agashivala N, Allen RR, Ghushchyan V, Yadao AM, Nair KV: Comparison of patient characteristics and gout-related health-care resource utilization and costs in patients with frequent versus infrequent gouty arthritis attacks. Rheumatology (Oxford) 2012, 51(11):2004–2012. 10.1093/rheumatology/kes183 22829689

[10] Wu EQ, Patel PA, Yu AP, Mody RR, Cahill KE, Tang J, Krishnan E (2008). Disease-related and all-cause health care costs of elderly patients with gout. J Manag Care Pharm.

[11] Sarawate CA, Brewer KK, Yang W, Patel PA, Schumacher HR, Saag KG, Bakst AW (2006). Gout medication treatment patterns and adherence to standards of care from a managed care perspective. Mayo Clin Proc.

[12] Harrold LR, Andrade SE, Briesacher BA, Raebel MA, Fouayzi H, Yood RA, Ockene IS (2009). Adherence with urate-lowering therapies for the treatment of gout. Arthritis Res Ther.

[13] Singh JA, Hodges JS, Asch SM (2009). Opportunities for improving medication use and monitoring in gout. Ann Rheum Dis.

[14] Singh JA, Hodges JS, Toscano JP, Asch SM (2007). Quality of care for gout in the US needs improvement. Arthritis Rheum.

[15] Singh JA, Sarkin A, Shieh M, Khanna D, Terkeltaub R, Lee SJ, Kavanaugh A, Hirsch JD (2011). Health care utilization in patients with gout. Semin Arthritis Rheum.

[16] Harrold LR, Saag KG, Yood RA, Mikuls TR, Andrade SE, Fouayzi H, Davis J, Chan KA, Raebel MA, Von Worley A (2007). Validity of gout diagnoses in administrative data. Arthritis Rheum.

[17] Malik A, Dinnella JE, Kwoh CK, Schumacher HR (2009). Poor validation of medical record ICD-9 diagnoses of gout in a veterans affairs database. J Rheumatol.

[18] Singh JA (2013). Veterans Affairs databases are accurate for gout-related health care utilization: a validation study. Arthritis Res Ther.

[19] Wallace SL, Robinson H, Masi AT, Decker JL, McCarty DJ, Yu TF (1977). Preliminary criteria for the classification of the acute arthritis of primary gout. Arthritis Rheum.

[20] Hirsch JD, Terkeltaub R, Khanna D, Singh J, Sarkin A, Shieh M, Kavanaugh A, Lee SJ (2010). Gout disease-specific quality of life and the association with gout characteristics. Patient Relat Outcome Meas.

[21] Hirsch JD, Lee SJ, Terkeltaub R, Khanna D, Singh J, Sarkin A, Harvey J, Kavanaugh A (2008). Evaluation of an instrument assessing influence of Gout on health-related quality of life. J Rheumatol.

[22] Borowsky SJ, Cowper DC (1999). Dual use of VA and non-VA primary care. J Gen Intern Med.

[23] Fleming C, Fisher ES, Chang CH, Bubolz TA, Malenka DJ (1992). Studying outcomes and hospital utilization in the elderly. The advantages of a merged data base for Medicare and Veterans Affairs hospitals. Med Care.

[24] Wright SM, Daley J, Fisher ES, Thibault GE (1997). Where do elderly veterans obtain care for acute myocardial infarction: Department of Veterans Affairs or Medicare?. Health Serv Res.

[25] Hynes DM, Koelling K, Stroupe K, Arnold N, Mallin K, Sohn MW, Weaver FM, Manheim L, Kok L (2007). Veterans’ access to and use of Medicare and Veterans Affairs health care. Med Care.

[26] Coley K, Saul M, Pater K (2012). Relationship Between Race, Uric Acid Levels, Urate-Lowering Therapy and Resource Use in Patients with Gout. Arthritis Rheum.

[27] Krishnan E, Griffith C, Kwoh K (2005). Burden of Illness from Gout in Ambulatory Care in the United States. Arthritis Rheum.

[28] Pol LG, Thomas RK (1992). The Demography of Health and Health Care.

[29] Agha Z, Lofgren RP, Vanruiswyk JV, Layde PM (2000). Are patients at Veterans Affairs medical centers sicker? a comparative analysis of health status and medical resource use. Arch Intern Med.

