# Supplementary material for: Health care utilization in patients with gout: a prospective multicenter cohort study
Source: BMC Musculoskelet Disord. 2017 May 31;18:233. doi: 10.1186/s12891-017-1573-6 (PMC5452408; doi:10.1186/s12891-017-1573-6)
Supplement: Supplementary file 1 — University of California at San Diego (UCSD) health care utilization questionnaire. Description: This file shows the copyrighted UCSD health care utilization questionnaire. (DOCX 992 kb) [file 12891_2017_1573_MOESM1_ESM.docx]

**Supplementary File**

**Additional file 1. University of California at San Diego (UCSD) health care utilization questionnaire**
