# Supplementary material for: Health care utilization in patients with gout: a prospective multicenter cohort study
Source: BMC Musculoskelet Disord. 2017 May 31;18:233. doi: 10.1186/s12891-017-1573-6 (PMC5452408; doi:10.1186/s12891-017-1573-6)
Supplement: Supplementary file 2 — Baseline Comorbidity Characteristics. Description: This file shows the detailed baseline comorbidity characteristics for the entire cohort and by each site for the study cohort. (DOCX 29 kb) [file 12891_2017_1573_MOESM2_ESM.docx]

**Additional file 2. Baseline Comorbidity Characteristics**

| Charlson Comorbidity | Total Cohort  N=186  N (%) | Los Angeles  N=112  N (%) | Birmingham  N=74  N (%) | Los Angeles vs. Birmingham  p-value |
| --- | --- | --- | --- | --- |
| Congestive heart failure |  |  |  |  |
| *Yes* | 27 (15.43) | 18 (16.22) | 9 (14.06) | 0.70 ^ε^ |
| *No* | 148 (84.57) | 93 (83.78) | 55 (85.94) |  |
| *Missing* | 11 | 1 | 10 |  |
| Myocardial infarction |  |  |  |  |
| *Yes* | 30 (17.05) | 19 (16.96) | 11 (17.19) | 0.97 ^ε^ |
| *No* | 146 (82.95) | 93 (83.04) | 53 (82.81) |  |
| *Missing* | 10 | 0 | 10 |  |
| Peripheral vascular disease |  |  |  |  |
| *Yes* | 20 (11.36) | 16 (14.29) | 4 (6.25) | 0.14 ^ε^ |
| *No* | 163 (92.61) | 96 (85.71) | 60 (93.75) |  |
| *Missing* | 10 | 0 | 10 |  |
| Cerebrovascular disease |  |  |  |  |
| *Yes* | 13 (7.39) | 13 (11.61) | 0 (0) | 0.003^β^ |
| *No* | 163 (92.61) | 99 (88.39) | 64 (100) |  |
| *Missing* | 10 | 0 | 10 |  |
| Dementia |  |  |  |  |
| *Yes* | 2 (1.13) | 2 (1.79) | 0 (0) | 0.53 ^β^ |
| *No* | 175 (98.87) | 110 (98.21) | 65 (100) |  |
| *Missing* | 9 | 0 | 9 |  |
| Chronic pulmonary disease |  |  |  |  |
| *Yes* | 28 (15.82) | 15 (13.39) | 13 (20.0) | 0.24 ^ε^ |
| *No* | 149 (84.18) | 97 (86.61) | 52 (80.0) |  |
| *Missing* | 9 | 0 | 9 |  |
| Connective tissue disease |  |  |  |  |
| *Yes* | 3 (1.70) | 3 (2.68) | 0 (0) | 0.55 ^β^ |
| *No* | 173 (98.30) | 109 (97.32) | 64 (100) |  |
| *Missing* | 10 | 0 | 10 |  |
| Ulcer disease |  |  |  |  |
| *Yes* | 22 (12.50) | 22 (19.64) | 0 (0) | <0.001^β^ |
| *No* | 154 (87.50) | 90 (80.36) | 64 (100) |  |
| *Missing* | 10 | 0 | 10 |  |
| Mild liver disease |  |  |  |  |
| *Yes* | 10 (5.71) | 9 (8.18) | 1 (1.54) | 0.09 ^β^ |
| *No* | 165 (94.29) | 101 (91.82) | 64 (98.46) |  |
| *Missing* | 11 | 2 | 9 |  |
| Diabetes |  |  |  |  |
| *Yes* | 64 (36.16) | 46 (41.07) | 18 (27.69) | 0.07 ^ε^ |
| *No* | 113 (63.84) | 66 (58.93) | 47 (72.31) |  |
| *Missing* | 9 | 0 | 9 |  |
| Depression |  |  |  |  |
| *Yes* | 54 (30.51) | 39 (34.82) | 15 (23.08) | 0.10 ^ε^ |
| *No* | 123 (69.49) | 73 (65.18) | 50 (76.92) |  |
| *Missing* | 9 | 0 | 9 |  |
| Hypertension |  |  |  |  |
| *Yes* | 144 (81.36) | 98 (87.50) | 46 (70.77) | 0.006 ^ε^ |
| *No* | 33 (18.64) | 14 (12.50) | 19 (29.23) |  |
| *Missing* | 9 | 0 | 9 |  |
| Hemiplegia |  |  |  |  |
| *Yes* | 8 (4.52) | 2 (1.79) | 6 (9.23) | 0.05 ^β^ |
| *No* | 169 (95.48) | 110 (98.21) | 59 (90.77) |  |
| *Missing* | 9 | 0 | 9 |  |
| Moderate or severe renal disease |  |  |  |  |
| *Yes* | 43 (24.43) | 24 (21.43) | 19 (29.69) | 0.22 ^ε^ |
| *No* | 133 (75.57) | 88 (78.57) | 45 (70.31) |  |
| *Missing* | 10 | 0 | 10 |  |
| Diabetes with end organ damage |  |  |  |  |
| *Yes* | 21 (11.86) | 20 (17.86) | 1 (1.54) | <0.001^β^ |
| *No* | 156 (88.14) | 92 (82.14) | 64 (98.46) |  |
| *Missing* | 9 | 0 | 9 |  |
| Any tumor |  |  |  |  |
| *Yes* | 28 (15.82) | 14 (12.50) | 14 (21.54) | 0.11 ^ε^ |
| *No* | 149 (84.18) | 98 (87.50) | 51 (78.46) |  |
| *Missing* | 9 | 0 | 9 |  |
| Leukemia |  |  |  |  |
| *Yes* | 0 (0) | 0 (0) | 0 (0) |  |
| *No* | 177 (100) | 112 (100) | 65 (100) |  |
| *Missing* | 9 | 0 | 9 |  |
| Lymphoma |  |  |  |  |
| *Yes* | 0 (0) | 0 (0) | 0 (0) |  |
| *No* | 177 (100) | 112 (100) | 65 (100) |  |
| *Missing* | 9 | 0 | 9 |  |
| Moderate or severe liver disease |  |  |  |  |
| *Yes* | 0 (0) | 0 (0) | 0 (0) |  |
| *No* | 177 (100) | 112 (100) | 65 (100) |  |
| *Missing* | 9 | 0 | 9 |  |
| Metastatic cancer |  |  |  |  |
| *Yes* | 0 (0) | 0 (0) | 0 (0) |  |
| *No* | 177 (100) | 112 (100) | 65 (100) |  |
| *Missing* | 9 | 0 | 9 |  |
| AIDS |  |  |  |  |
| *Yes* | 2 (1.14) | 1 (0.9) | 1 (1.54) | >0.99^β^ |
| *No* | 174 (98.86) | 110 (99.1) | 64 (98.46) |  |
| *Missing* | 10 | 1 | 9 |  |

α=T-test; β=Fisher Exact test; γ=Wilcoxon test; ε=Chi-square test

LA, Los Angeles; BHAM, Birmingham
