# Supplementary material for: Health care utilization in patients with gout: a prospective multicenter cohort study
Source: BMC Musculoskelet Disord. 2017 May 31;18:233. doi: 10.1186/s12891-017-1573-6 (PMC5452408; doi:10.1186/s12891-017-1573-6)
Supplement: Supplementary file 3 — Unadjusted estimates of overall healthcare utilization and gout-specific utilization. Description: This file shows the unadjusted estimates of overall healthcare utilization and for gout-specific utilization in the study cohort, detailed by the type of healthcare utilization. (DOCX 18 kb) [file 12891_2017_1573_MOESM3_ESM.docx]

**Additional file 3. Unadjusted estimates of overall healthcare utilization and gout-specific utilization**

|  | Patients with at least one visit/call | | Number of visits/calls | |
| --- | --- | --- | --- | --- |
|  | N | % | Mean | SD |
| Overall healthcare utilization, prior 3 months: |  |  |  |  |
| Number of visits to MD, DO, or NP | 163 | 92% | 3.9 | 4.3 |
| Number of phone calls to MD or medical staff | 85 | 48% | 1.7 | 3.1 |
| Number of times to a triage, urgent care center, or emergency room | 74 | 41% | 0.6 | 0.9 |
| Number of home visits by healthcare provider | 16 | 9% | 0.6 | 2.4 |
| Number of days as inpatient | 31 | 17% | 1.8 | 9.6 |
| Number of outpatient surgeries or procedures | 27 | 16% | 0.3 | 0.9 |
|  |  |  |  |  |
| Gout-related healthcare utilization in the prior year: |  |  |  |  |
| Rheumatologist | 97 | 53% | 1.5 | 2 |
| Primary Care Physician | 128 | 71% | 2 | 1.9 |
| Nurse Practitioner or Physician Assistant | 53 | 31% | 0.8 | 1.6 |
| Walk-in or Urgent Care Clinic | 57 | 33% | 0.7 | 1.5 |
| Emergency Room at a hospital | 45 | 26% | 0.4 | 0.8 |
| Hospital Over-night Stay | 17 | 10% | 0.7 | 7.5 |

SD, standard deviation; MD, physician; DO, doctor of osteopathy; NP, nurse practitioner
