# Supplementary material for: Health care utilization in patients with gout: a prospective multicenter cohort study
Source: BMC Musculoskelet Disord. 2017 May 31;18:233. doi: 10.1186/s12891-017-1573-6 (PMC5452408; doi:10.1186/s12891-017-1573-6)
Supplement: Supplementary file 4 — Cross-sectional Multivariable-adjusted predictors of gout-related outpatient and Urgent/emergent/overnight visits in the 3 months prior to the baseline. Description: This file shows the multivariable-adjusted analyses using the cross-sectional data for both gout-related outpatient and non-outpatient healthcare utilization. (DOCX 18 kb) [file 12891_2017_1573_MOESM4_ESM.docx]

**Additional file 4. Cross-sectional Multivariable-adjusted predictors of gout-related outpatient and Urgent/emergent/ overnight** **visits in the three months before the baseline visit**

|  | Incidence rate ratio  (95% CI) | P-value |
| --- | --- | --- |
| **Gout-related Outpatient visits** | | |
| Age  (for every 1 year increase) | **1.01**  **(1.00, 1.02)** | **0.02** |
| African-American  (Ref, Caucasian) | **1.38**  **(1.14, 1.67)** | **<0.01** |
| College Education  (Ref, no college education) | **1.57**  **(1.28, 1.93)** | **<0.01** |
| Baseline sUA  (for every 1 mg/dl increase) | 0.98  (0.96, 1.01) | 0.13 |
| Charlson Index  (for every unit increase) | **0.94**  **(0.90, 0.97)** | **<0.01** |
| Gout Attack in Past 3-months  (Ref, no attack) | **1.86**  **(1.50, 2.30)** | **<0.01** |
| **Gout-related Urgent/emergent/ overnight visits** | | |
| Age  (for every 1 year increase) | 1.00  (0.98, 1.02) | 0.91 |
| African-American  (Ref, Caucasian) | **2.06**  **(1.47, 2.88)** | **<0.01** |
| College Education  (Ref, no college education) | 1.21  (0.84, 1.74) | 0.30 |
| Baseline sUA  (for every 1 mg/dl increase) | 1.02  (0.98, 1.07) | 0.27 |
| Charlson Index  (for every unit increase) | 0.99  (0.93, 1.05) | 0.79 |
| Gout Attack in past 3-months  (Ref, no attack) | **2.33**  **(1.53, 3.53)** | **<0.01** |

* Includes walk-in-urgent-care-visits, emergency-room-visits, hospital-over-night-visits

Significant Incidence rate ratios are **in bold**

Ref, Reference category
